# A Cross Sectional Study on Serological Prevalence of *Ehrlichia canis* and *Rickettsia* *conorii* in Different Canine Population of Sicily (South-Italy) during 2017–2019

**DOI:** 10.3390/ani10122444

**Published:** 2020-12-20

**Authors:** Sergio Migliore, Valeria Gargano, Claudia De Maria, Delia Gambino, Antonino Gentile, Valeria Vitale Badaco, Giorgia Schirò, Francesco Mira, Paola Galluzzo, Domenico Vicari, Santina Di Bella

**Affiliations:** 1 Istituto Zooprofilattico Sperimentale della Sicilia “A. Mirri”, 90129 Palermo, Italy; sergio.migliore@izssicilia.it (S.M.); cdemariavet@gmail.com (C.D.M.); deliagamb@gmail.com (D.G.); antogentile1980@gmail.com (A.G.); valeria.badaco@izssicilia.it (V.V.B.); giorgia.schiro91@gmail.com (G.S.); dottoremira@gmail.com (F.M.); paola.galluzzo@izssicilia.it (P.G.); domenico.vicari@izssicilia.it (D.V.); santina.dibella@izssicilia.it (S.D.B.); 2 Dipartimento Scienze e Tecnologie Biologiche, Chimiche e Farmaceutiche, Viale delle Scienze, University of Palermo, 90128 Palermo, Italy

**Keywords:** *Erlichia canis*, *Rickettsia conorii*, dog

## Abstract

**Simple Summary:**

Our study provides a picture of *Ehrlichia canis* and *Rickettsia conorii* seroprevalence in Sicilian dogs, in the period from 2017 to 2019. *E. canis* and *R. conorii* are canine, vector-borne pathogens widespread in the Mediterranean basin infecting humans and a wide variety of domestic and wild animals. The aim of this work was to evaluate the presence of antibody against these two pathogens in dogs and confirm their wide distribution in Sicily. In this study, we reported a prevalence of 29.6% and 53.6% for *E. canis* and *R. conorii*, respectively, confirming the widespread distribution of these pathogens in our territory. Temporal variation was found only in *R conorii* infection, with the highest prevalence (60.6%) reported during 2018. Regarding the spatial variation, the significant difference of seroprevalence was found comparing the climate areas. In particular, the least rainy area showed the higher seroprevalence for both infections. The obtained results suggest that *E. canis* and *R. conorii* are present in Sicily in different areas and canine population. Prevention and surveillance of the entire canine population remain the main tools for preventing infection and identifying the areas most exposed to risk.

**Abstract:**

Vector-borne pathogens such as *Erlichia canis* and *Rickettsia conorii* are widespread in the Mediterranean basin. *Rhipicephalus sanguineus*, is considered the main vector in Mediterranean climatic areas. Seroprevalence in dogs is variable in relation to environmental factors, presence of vectors, and dogs’ management. We investigated the seroprevalence in Sicilian dogs during 2017–2019, considering temporal as well as spatial variations, and different canine population. A total of 11,009 sera were analyzed: 7568 and 3441 sera were tested to detect antibodies to *E. canis* and to *R. conorii*, respectively, by immunofluorescence assay. The rainfall average in the sampling sites during the three-year period was also considered. Statistical analyses were performed using chi-square tests for association between two or more variables. We reported a prevalence of 29.6% and 53.6% for *E. canis* and *R. conorii,* respectively. Significant temporal variation was found in *R. conorii*, while significant difference was found considering canine population and spatial variation regarding both pathogens. Our study updates the previous results of *E. canis* and *R. conorii* seroprevalence in dogs in Sicily, and confirms the wide distribution of these pathogens. In addition, we considered, for the first time, three different variables to identify the areas and the canine populations most exposed to risk.

## 1. Introduction

Canine vector-borne pathogens such as *Ehrlichia canis* and *Rickettsia conorii* are obligate intracellular coccoid and Gram-negative organisms belonging to the *Rickettsiales* order. These pathogens infecting humans and a wide variety of domestic and wild animals [1] are widespread in the Mediterranean basin. Generally, they are species-specific host and some hosts might play a reservoir role for infection [2]. Dogs are the specific hosts of *E. canis* but infection has been also described in cats and other canids [3,4], for which a zoonotic role has been supposed [5]. In dogs, *E. canis* is the organism responsible for the canine monocytic ehrlichiosis (CME). *R. conorii* infection, responsible for Mediterranean spotted fever, is recognized as the most important zoonotic agents of the *Rickettsia* genus in Mediterranean countries, Sub Saharan Africa and Asia, where dogs are considered the sentinel of the infection and the natural host [6]. The presence of *R. conorii* in cats was also reported [7,8]. *E. canis* and *R. conorii* are vector-borne pathogens transmitted by ticks during their blood meal. *Rhipicephalus sanguineus* sensu lato (*s.l.*), known as the brown dog-tick, is considered the main vector of *E. canis* and *R. conorii* in Europe [9]. *R. sanguineus* is widely present in Italy [10] and in the Mediterranean basin, and requires a good degree of humidity and a minimum environmental temperature of 6 °C. These ticks are able to hibernate when environmental temperatures are too low [11]. Climate changes and anthropogenic factors have influenced the distribution of Canine vector-borne pathogens worldwide during recent decades [12]. Serological prevalence of these pathogens in dogs are very variable in relation to environmental factors such as geographic area, climate condition, and presence of vectors. In addition, available data are strictly related to hosts features (attitude, free-roaming or owned, kennel dog) and its relative infection exposure (prophylaxis against the vectors, management, and environment).

Climatic conditions may influence the spreading of ticks and consequently the presence of tick-borne pathogens in animal populations. Each tick species that may act as a vector favors particular optimal environmental conditions and biotopes. These determine the geographic distribution of the ticks and consequently the risk area for tick-borne diseases [13]. 

Sicily is the largest island in the Mediterranean basin and represents a typical Mediterranean ecosystem for this tick [14]. Nevertheless, in Italy the Istituto Superiore per la Protezione e la Ricerca Ambientale (I.S.P.R.A.) reported a climate change in recent decades [15]. In particular, the 2018 average temperature value in Italy was the highest since 1961. Specifically, the average annual temperature was 1.33 °C higher in South-Italy, with a maximum increase of 3.12 °C in April. In addition, the average annual rainfall in 2018 in South Italy, was 29% above average, compared to the three decades period 1961–1990, with an increase of 275% in August, 226% in June and 132% in May [16]. This new climatic scenario suggests an extension of ticks’ activity period in Southern Italy from April to November, which increases the natural host exposure to tick-borne pathogens. 

Therefore, the aim of the present study was to investigate the overall serological prevalence of *E. canis,* and *R. conorii* in dogs in Sicily (South-Italy) during the three-year period, 2017–2019, in relation to temporal (annual) and spatial (climatic areas) variations, and canine population (owned and shelter dogs).

## 2. Materials and Methods

### 2.1. Ethical Statement

The study did not involve any animal experiments. Blood samples were taken from dogs that were naturally infected or in which tick-borne disease was suspected. Blood sampling was necessary inorder to perform laboratory analysis and did not involve any suffering of the animals sampled.

### 2.2. Study Area

Samples were collected in Sicily, Italy. In this region, the climate is Mediterranean along the coasts and on the smaller islands; Winter is mild with rare or absent snow and frost, while summer is hot and sunny, with temperature often exceeding 35 °C. In inland areas, the climate is slightly more continental on the hills, with a strong seasonal and daily temperature range. Summer is still hot, while frosts and snowfalls are common in winter with values that also drop below 0 °C.

**Figure 1:**
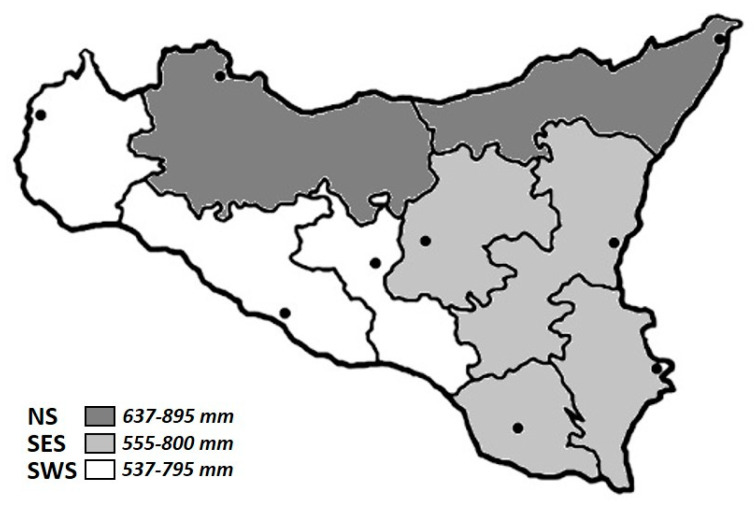
Study areas depending on average rainfall during the considered period (2017–2019).

We divided the sampling sites into three main areas (Figure 1), depending on average rainfall during the considered period (2017–2019) [16]:

- Northern Sicily (NS): includes the Tyrrhenian side of the island, in particular Palermo and Messina districts. Rainfall is characterized by a rainy season (autumn–winter) and a dry spring–summer (rainy days per year > 70).
- South-Eastern Sicily (SES): includes Catania, Syracuse, Ragusa and Enna. Rainfall is usually less frequent than in the Tyrrhenian area and the rainy days do not exceed 60.
- South-Western Sicily (SWS): includes the area bordered by the Mediterranean, the Sicilian Channel and the central area: Trapani, Agrigento and Caltanissetta districts. The number of rainy days is lower than other areas (<60 days per year).

### 2.3. Sample

A total of 11,009 dog sera were included in this study during three year period, from January 2017 to December 2019. From the total number, 7568 and 3441 sera were tested by immunofluorescence assays to detect antibodies to *E. canis* and to *R. conorii*, respectively. According to the geographic areas, the sample was divided into three main groups: NS = 5547 (50.4%), SES = 2535 (23%), and SEW = 2927 (26.6%) samples. For each group, we stratified the samples in two categories according to the dogs’ population: shelter dogs (S) and owned dogs (O) (Table 1). Samples of shelter dogs were collected immediately after they were admitted to the shelter for health reasons, for spaying or for future adoption. Information about age, gender, clinical status, or tick infestation were not available.

### 2.4. Serological Analysis 

Tested samples were part of the routine and scientific activity of the National Reference Center for *Anaplasma*, *Babesia*, *Rickettsia* and *Theileria* (C.R.A.Ba.R.T.), situated at the Istituto Zooprofilattico Sperimentale della Sicilia “A. Mirri” (Italy). Whole-blood samples were centrifuged at 1500× *g* for 15 min and then serum was separated from the clot. The sera were collected and immediately tested or stored at −20 °C. All sera were examined by indirect immunofluorescence assay (IFA) to detect antibodies to *E. canis* and *R. conorii* using *Erlichia canis* IgG IFA kit (Fuller Laboratories—Fullerton, CA, USA) and Canine *Rickettsia conorii* IgG IFA kit (Fuller Laboratories—Fullerton, CA, USA), respectively, according to the manufacturer’s instructions. Samples with titres of 1:50, and of 1:64 or greater for *E. canis* and *R. conorii*, respectively, were considered as positive.

### 2.5. Statistical Analysis

Statistical analyses were performed using chi-square function in R software [17] and Bonferroni correction was applied when three groups were compared. Proportion differences between temporal, spatial, and group variations were tested. Values of *p* < 0.05 were considered significant and, after Bonferroni correction, a *p*-value of 0.016 was considered. The null hypothesis asserts no differences among groups.

## 3. Results

From the total number of 11,009 dog sera collected in Sicily, the 68.7% (7568 dogs) were tested for *E. canis* while the 31.3% (3441 dogs) for *R. conorii* infection. The highest prevalence of both pathogens was recorded in 2018, with 30.4% of positive for *E. canis* and with 60.6% of positive for *R. conorii*. In 2017 the lowest prevalence of *E. canis* was revealed, while in 2019 the lowest prevalence of *R. conorii* was detected.

The overall prevalence of *E. canis* infection during 2017–2019 was 29.6%, with no annual statistical differences detected (Table 2 and Supplementary Materials Table S1). 

Compared to 7568 analyzed samples, 40% originated from NS area and 30% from SES and SWS areas, respectively. Considering the study areas, we found a prevalence of 25.4% in NS, 31.6% in SES and 33.3% in SWS (Table 3). 

Significant statistical difference (*p* < 0.016) was found comparing NS with SES and SWS areas (Supplementary Materials Table S2). Within the NS groups, we found 25.8% and 24.1% of seropositive dogs in S and O, respectively. In SWS area, we found 33.5% of positive in S and 32.3% in O groups (Table 4 and Supplementary Materials Table S3). 

No statistical differences were found within these subgroups. In the SES area, we observed a prevalence of 32.5% in S and 20.1% in O subgroups, respectively. This result is statistically significant with *p* < 0.05.

The overall prevalence of *R. conorii* infection during the three year study period was 53.6%. A statistically significant difference between the years was detected (*p* < 0.016) (Table 2 and Supplementary Materials Table S4).

Compared to 3341 tests for *R. conorii*, 71% originated from NS, 9% from SES, and 20% from SEW areas. We found a prevalence of 52.1% in NS, 42% in SES, and 64.1% in SEW (Table 3). Statistical differences between the groups were detected (*p* < 0.05) (Supplementary Materials Table S5). Within the NS group, we found 50% of positive dogs in S and 58.5% in O subgroups, respectively. In SES area, we found 60% of positive in S and 69.1% in O subgroups. In SES area, we observed a prevalence of 45.2% in S and 29.5% in O subgroups, respectively (Table 4). Statistical differences between S and O categories in each group are significant at *p* < 0.05 (Supplementary Materials Table S6).

## 4. Discussion

Sicily represents a typical Mediterranean ecosystem to study tick infestations and the prevalence of endemic tick-borne pathogens [14]. In Sicily, many tick-borne diseases are endemic, in particular those transmitted by carrier ticks that prefer, for their vital cycle, climatic conditions characterized by high temperatures and a warmth-humid atmosphere [18]. 

Strays, as hosts of zoonotic pathogens, could represent a potential threat for human health. In Sicily, a high population of stray dogs in urban and peri urban areas is present [19], and this represents a known but yet underestimated problem [20]. Official data on the free-roaming stray population is not available. However, the Lega Anti Vivisezione (LAV), a non-governmental animal rights association, conducted a census in 74 shelters located in Sicily in 2017, and reported that 13,185 dogs were housed in these shelters; of these, 8673 dogs were recovered in the same year, with an increase of 22% over the previous year and of 28% compared to 2006 [21].

Our research centre, C.R.A.Ba.R.T, conducted many studies and carried out many tests with diagnostic aim in order to estimate the spread of the main tick borne diseases in Sicilian dogs. A previous study on 342 dogs carried out in the two year period 2004–2005, reported the presence of *E. canis* and *R. conorii* in all Sicilian areas with an overall seroprevalence of 21.70% and 53.43%, respectively [18]. Another study performed in Sicily, on 249 outdoor-kenneled dogs in the limited area of Strait of Messina, compared the seroprevalence in two public shelters and four privately-owned kennels where different tick-preventive measures were implemented. *R. conorii* infection showed a high seroprevalence (72%) compared with *E. canis* (46%) that was significantly higher in public shelters than in private kennels [22]. The two pathogens seroprevalence found in our study is similar with that was reported in the north of Spain by Solano-Gallego et al. (56.4% for *R. conorii* and 16.7% for *E. canis*) [23]. A big difference, instead, can be found with the data relating to northern Italy reported by Vascellari et al., according to which *R. conorii* was present in 21.3% of the analyzed dogs and *E. canis* in 0.9% [24].

In this study, we reported an update of the seroprevalence of *E. canis* and *R. conorii* infection in the overall canine population in Sicily during three year period (2017–2019). We considered three variables, to better understand the factors that may have influenced spread of the main vector-borne pathogens within the canine population of Sicily. We found an overall prevalence of 29.6% and 53.6% for *E. canis* and *R. conorii* infection, respectively. These prevalence results were lower compared to the dogs sampled in the area of Messina Strait in 2012. Regarding *E. canis* infection, we reported a seroprevalence that was higher than the overall Sicilian canine population reported in 2004–2005. This is in contrast to the similar seroprevalence described for *R. conorii* during the same period. Our results add interesting information to the previous study. Temporal variation was found only in *R conorii* infection, with the highest prevalence (60.6%) reported during 2018, the rainiest and warmest year under examination (Table 2). Regarding the spatial variation, significant difference of seroprevalence was found compared the climate areas, in particular in the least rainy area (SWS) that showed the highest seroprevalence for both infections (Table 3). These results suggest that climatic and environmental conditions in the area bordered by the Mediterranean might favour an extension of ticks activity period. Significant differences were also reported comparing the dogs’ population. In some cases, seroprevalence was higher in the owned dogs (Table 4). This could be due to a greater attention of the owner who seeks for a veterinary consultation following the appearance of clinical symptoms.

Unfortunately, only limited data on seroprevalence of these tick-borne infections in canine population of Mediterranean basin are available and it is very difficult to compare the results, mainly due to the different climatic micro-areas, different vector species and different canine population. 

## 5. Conclusions

Despite the increased sensitivity of owners to tick-borne diseases in recent years, our results confirm that *E. canis* and *R. conorii* are still present in Sicily. The prevention of tick infestation in the entire canine population, combined with constant surveillance of the territory, remain the main tool for preventing infections and identifying the areas most exposed to risk.

## Figures and Tables

**Table 1:** Sample categories according to geographic areas defined in the study: Northern Sicily (NS), South Western Sicily (SWS), South Eastern Sicily (SES) and dogs’ population, shelter dogs (S) and owned dogs (O).

Groups	*E. canis*		*R. conorii*	
	S	O	S	O
NS	2327	773	1849	598
SWS	1866	374	380	307
SES	2064	164	246	61

**Table 2:** Seroprevalence of *E. canis* and *R. conorii* among tested sera in relationship to annual variation.

year	*E. canis*		*R. conorii*	
	No. of Sera	Positive (%)	No. of Sera	Positive (%)
2017	2359	678 (28.7)	872	481 (55.2)
2018	2553	775 (30.4)	1.312	795 (60.6)
2019	2656	785 (29.6)	1.257	576 (45.1)
total	7568	2238 (29.6)	3441	1843 (53.6)

**Table 3:** Serological results in relationship to spatial variations: Northern Sicily (NS), South Western Sicily (SWS), and South Eastern Sicily (SES).

Groups	*E. canis*		*R. conorii*	
	No. of Sera	Positive (%)	No. of Sera	Positive (%)
NS	3100	787 (25.4)	2.447	1274 (52.1)
SWS	2240	747 (33.3)	687	440 (64.1)
SES	2228	704 (31.6)	307	129 (42.0)

**Table 4:** Serological results in relationship to dog population: Shelter (S) or owned (O) dogs.

Groups	Categories	*E. canis*		*R. conorii*	
		No. of Sera	Positive (%)	No. of Sera	Positive (%)
NS	S	2327	601 (25.8)	1849	924 (50.0)
	O	773	186 (24.1)	598	350 (58.5)
SWS	S	1866	626 (33.5)	380	228 (60.0)
	O	374	121 (32.4)	307	212 (69.1)
SES	S	2064	671 (32.5)	246	111 (45.1)
	O	164	33 (20.1)	61	18 (29.5)

## Supplementary Materials

The following are available online at https://www.mdpi.com/2076-2615/10/12/2444/s1. Table S1: Temporal difference in *E. canis* infection. Table S2: Spatial differences in *E. canis* infection. Table S3: Difference between *E. canis* infection in the several groups analysed. Table S4: temporal difference in *R. conorii* infection. Table S5: Spatial differences in *R. conorii* infection. Table S6: Difference between *R. conorii* infection in the several groups analysed.

## References

[1] Allison R.W., Little S.E. (2013). Diagnosis of rickettsial diseases in dogs and cats. Vet. Clin. Pathol..

[2] Pennisi M.G., Hofmann-Lehmann R., Radford A.D., Tasker S., Belák S., Addie D.D., Boucraut-Baralon C., Egberink H., Frymus T., Gruffydd-Jones T. (2017). *Anaplasma, Ehrlichia* and *Rickettsia* species infections in cats: European guidelines from the ABCD on prevention and management. Feline Med. Surg..

[3] Maia C., Ramos C., Coimbra M., Bastos F., Martins A., Pinto P., Nunes M., Vieira M.L., Cardoso L., Campino L. (2014). Bacterial and protozoal agents of feline vector-borne diseases in domestic and stray cats from southern Portugal. Parasites Vectors.

[4] Ebani V.V., Rocchigiani G., Nardoni S., Bertelloni F., Vasta V., Papini R.A., Verin R., Poli A., Mancianti F. (2017). Molecular detection of tick-borne pathogens in wild red foxes (*Vulpes vulpes*) from Central Italy. Acta Trop..

[5] Perez M., Bodor M., Zhang C., Xiong Q., Rikihisa Y. (2006). Human infection with *Ehrlichia canis* accompanied by clinical signs in Venezuela. Ann. N. Y. Acad. Sci..

[6] Parola P., Paddock C.D., Raoult D. (2005). Tick-borne rickettsioses around the world: Emerging diseases challenging old concepts. Clin. Microbiol. Rev..

[7] Persichetti M.F., Pennisi M.G., Vullo A., Masucci M., Migliazzo A., Solano-Gallego L. (2018). Clinical evaluation of outdoor cats exposed to ectoparasites and associated risk for vector-borne infections in southern Italy. Parasites Vectors.

[8] Morganti G., Veronesi F., Stefanetti V., Di Muccio T., Fiorentino E., Diaferia M., Santoro A., Passamonti F., Gramiccia M. (2019). Emerging feline vector-borne pathogens in Italy. Parasites Vectors.

[9] Sainz Á., Roura X., Miró G., Estrada-Peña A., Kohn B., Harrus S., Solano-Gallego L. (2015). Guideline for veterinary practitioners on canine ehrlichiosis and anaplasmosis in Europe. Parasites Vectors.

[10] Scarpulla M., Barlozzari G., Marcario A., Salvato L., Blanda V., De Liberato C., D’Agostini C., Torina A., Macrì G. (2016). Molecular detection and characterization of spotted fever group rickettsiae in ticks from Central Italy. Ticks Tick Borne Dis..

[11] Gray J., Dantas-Torres F., Estrada-Pena A., Levin M. (2013). Systematics and ecology of the brown dog tick, *Rhipicephalus sanguineus*. Ticks Tick Borne Dis..

[12] Beugnet F., Chalvet-Monfray K. (2013). Impact of climate change in the epidemiology of vector-borne diseases in domestic carnivores. Comp. Immunol. Microbiol. Infect. Dis..

[13] Parola P., Raoult D. (2001). Tick-borne bacterial diseases emerging in Europe. Clin. Microbiol. Infect..

[14] Torina A., Alongi A., Naranjo V., Estrada-Peña A., Vicente J., Scimeca S., Marino A.M.F., Salina F., Caracappa S., de la Fuente J. (2008). Prevalence and genotypes of *Anaplasma* species and habitat suitability for ticks in a Mediterranean ecosystem. Appl. Environ. Microbiol..

[15] ISPRA (2019). Gli Indicatori del CLIMA in Italia nel 2018. https://www.isprambiente.gov.it/it/pubblicazioni/stato-dellambiente/gli-indicatori-del-clima-in-italia-nel-2018.

[16] Dipartimento Regionale dell’acqua e dei rifiuti—Osservatorio delle acque (2020). Personal communication.

[17] R Core Team (2014). A Language and Environment for Statistical Computing.

[18] Torina A., Caracappa S. (2006). Dog tick-borne diseases in Sicily. Parassitologia.

[19] Migliore S., La Marca S., Stabile C., Di Marco Lo Presti V., Vitale M. (2017). A rare case of acute toxoplasmosis in a stray dog due to infection of *T. gondii* clonal type I: Public health concern in urban settings with stray animals?. BMC Vet. Res..

[20] Galluzzo P., Grippi F., Di Bella S., Santangelo F., Sciortino S., Castiglia A., Sciacca C., Arnone M., Alduina R., Chiarenza G. (2020). Seroprevalence of *Borrelia burgdorferi* in Stray Dogs from Southern Italy. Microorganisms.

[21] LAV Randagismo: L’indagine LAV 2018. https://www.lav.it/cpanelav/js/ckeditor/kcfinder/upload/files/files/Dossier%20randagismo%202018.pdf.

[22] Pennisi M.G., Caprì A., Solano-Gallego L., Lombardo G., Torina A., Masucci M. (2012). Prevalence of antibodies against *Rickettsia conorii*, *Babesia canis*, *Ehrlichia canis*, and *Anaplasma phagocytophilum* antigens in dogs from the Stretto di Messina area Italy. Ticks Tick Borne Dis..

[23] Solano-Gallego L., Llull J., Osso M., Hegarty B., Breitschwerdt E. (2006). A serological study of exposure to arthropod-borne pathogens in dogs from northeastern Spain. Vet. Res..

[24] Vascellari M., Ravagnan S., Carminato A., Cazzin S., Carli E., Da Rold G., Lucchese L., Natale A., Otranto D., Capelli G. (2016). Exposure to vector-borne pathogens in candidate blood donor and free-roaming dogs of northeast Italy. Parasites Vectors.

