# Supplementary material for: A Cross Sectional Study on Serological Prevalence of Ehrlichia canis and Rickettsia conorii in Different Canine Population of Sicily (South-Italy) during 2017–2019
Source: Animals (Basel). 2020 Dec 20;10(12):2444. doi: 10.3390/ani10122444 (PMC7767218; doi:10.3390/ani10122444)
Supplement: Supplementary file 1 [file animals-10-02444-s001.pdf]

Table S1: Temporal difference in *E. canis* infection.

Year	Results		
	Positive	Negative	Row Totals
2017	678 (697.60) [0.55]	1681 (1661.40) [0.23]	2359
2018	775 (754.97) [0.53]	1778 (1798.03) [0.22]	2553
2019	785 (785.43) [0.00]	1871 (1870.57) [0.00]	2656
Column Totals	2238	5330	7568 (Grand Total)

The chi-square statistic is 1.5368. The *p*-value is 0.463746. The result is not significant at *p*<0.05. Bonferroni correction: Result is not significant at p<0.016

Table S2: Spatial differences in *E. canis* infection.

Groups	Results		
	Positive	Negative	Row Totals
NS	787 (894.03) [23.85]	2,313 (2166.97) [9.84]	3,061
SWS	747 (654.24) [13.15]	1,493 (1585.76) [5.43]	2,240
SES	704 (650.73) [4.36]	1,524 (1577.27) [1.80]	2,228
Column Totals	2,199	5,330	7529 (Grand Total)

The chi-square statistic is 58.4301. The *p*-value is < 0.00001. The result is significant at *p* <0.05. Bonferroni correction: Result is significant at p<0.016

Table S3: Difference between *E. canis* infection in the several groups analysed.

a. S vs O in NS

Groups	Results		
	Positive	Negative	Row Totals
NS/S	601 (589.87) [0.21]	1,712 (1723.13) [0.07]	2,313
NS/O	186 (197.13) [0.63]	587 (575.87) [0.22]	773
Column Totals	787	2,299	3,086 (Grand Total)

The chi-square statistic is 1.1259. The *p* -value is 0.288648. The result is not significant at *p*<0.05.

b. S vs O in SWS

Groups	Results		
	Positive	Negative	Row Totals
SWS/S	626 (622.28) [0.02]	1,240 (1243.72) [0.01]	1,866
SWS/O	121 (124.72) [0.11]	253 (249.28) [0.06]	374
Column Totals	747	1,493	2,240 (Grand Total)

The chi-square statistic is 0.2001. The *p* -value is 0.654655. The result is not significant at *p*<0.05.

c. S vs O in SES

Groups	Results		
	Positive	Negative	Row Totals
SES/S	671 (652.18) [0.54]	1,393 (1411.82) [0.25]	2,064
SES/O	33 (51.82) [6.84]	131 (112.18) [3.16]	164
Column Totals	704	1,524	2,228 (Grand Total)

The chi-square statistic is 10.7869. The *p* -value is 0.001022. The result is significant at *p*<0.05.

Table S4: temporal difference in *R. conorii* infection.

Year	Results		
	Positive	Negative	Row Totals
2017	481 (467.04) [0.42]	391 (404.95) [0.48]	872
2018	795 (702.71) [12.12]	517 (609.29) [13.98]	1,312
2019	567 (673.25) [16.77]	690 (583.75) [19.34]	1,257
Column Totals	1,843	1598	3,441 (Grand Total)

The chi-square statistic is 63.1061. The p-value is 0.00001. The result is significant at $p<0.05$. Bonferroni correction: Result is significant at p<0.016

Table S5: Spatial differences in *R. conorii* infection.

Groups	Results		
	Positive	Negative	Row Totals
NS	1274 (1287.26) [1.26]	1173 (1132.72) [1.43]	2420
SWS	440 (365.43) [15.21]	247 (311.28) [17.29]	687
SES	129 (163.30) [7.21]	178 (143.70) [8.19]	307
Column Totals	1,816	1,598	3134 (Grand Total)

The chi-square statistic is 50.5894. The p-value is < 0.00001. The result is significant at $p < 0.05$. Bonferroni correction: Result is significant at p<0.016

Table S6: Difference between *R. conorii* infection in the several groups analysed.

a. S vs PO in NS

Groups	Results		
	Positive	Negative	Row Totals
NS/S	924 (962.66) [1.55]	925 (886.34) [1.69]	1,849
NS/O	350 (311.34) [4.80]	248 (286.66) [5.21]	598
Column Totals	1,274	1173	2,447 (Grand Total)

The chi-square statistic is 13.2523. The p -value is 0.000272. The result is significant at $p<0.05$.

b. S vs PO in SWS

Groups	Results		
	Positive	Negative	Row Totals
SWS/S	228 (243.38) [0.97]	152 (136.62) [1.73]	380
SWS/O	212 (196.62) [1.20]	95 (110.38) [2.14]	307
Column Totals	440	247	687 (Grand Total)

The chi-square statistic is 6.047. The p -value is 0.01393. The result is significant at $p<0.05$.

c. S vs PO in SES

Groups	Results		
	Positive	Negative	Row Totals
SES/S	111 (103.37) [0.56]	135 (142.63) [0.41]	246
SES/O	18 (25.63) [2.27]	43 (35.37) [1.65]	61
Column Totals	129	178	307 (Grand Total)

The chi-square statistic is 4.8911. The p -value is 0.026995. The result is significant at $p<0.05$.
